# Generalist medical foundation model improves prostate cancer segmentation from multimodal MRI images

**DOI:** 10.1038/s41746-025-01756-2

**Published:** 2025-06-18

**Authors:** Yuhan Zhang, Xiao Ma, Mingchao Li, Kun Huang, Jie Zhu, Miao Wang, Xi Wang, Menglin Wu, Pheng-Ann Heng

**Affiliations:** 1 School of Biomedical Engineering, Shenzhen University, Shenzhen, China; 2 Department of Computer Science and Engineering, The Chinese University of Hong Kong, Hong Kong, China; 3 Department of Computer Science and Engineering, Nanjing University of Science and Technology, Nanjing, China; 4 Bioengineering Department and Imperial-X, Imperial College London, London, UK; 5 Academy of Arts and Design, Tsinghua University, Beijing, China; 6 Senior Department of Urology, The Third Medical Center of Chinese PLA General Hospital, Beijing, China; 7 Department of Urology, Beijing Hospital, National Center of Gerontology, Institute of Geriatric Medicine, Chinese Academy of Medical Sciences, Beijing, China; 8 Department of Computer Science and Engineering, The Hong Kong University of Science and Technology, Hong Kong, China; 9 Carbon Medical Device Ltd., Shenzhen, China; 10 School of Computer Science and Technology, Nanjing Tech University, Nanjing, China; 11 Institute of Medical Intelligence and XR, The Chinese University of Hong Kong, Hong Kong, China

**Keywords:** Cancer screening, Cancer imaging

## Abstract

Prostate cancer (PCa) is one of the most common types of cancer, seriously affecting adult male health. Accurate and automated PCa segmentation is essential for radiologists to confirm the location of cancer, evaluate its severity, and design appropriate treatments. This paper presents PCaSAM, a fully automated PCa segmentation model that allows us to input multi-modal MRI images into the foundation model to improve performance significantly. We collected multi-center datasets to conduct a comprehensive evaluation. The results showed that PCaSAM outperforms the generalist medical foundation model and the other representative segmentation models, with the average DSC of 0.721 and 0.706 in the internal and external datasets, respectively. Furthermore, with the assistance of segmentation, the PI-RADS scoring of PCa lesions was improved significantly, leading to a substantial increase in average AUC by 8.3–8.9% on two external datasets. Besides, PCaSAM achieved superior efficiency, making it highly suitable for real-world deployment scenarios.

## Introduction

Prostate cancer (PCa) is a significant health concern, being one of the most prevalent cancers among men globally^1^. It involves the abnormal growth of cells in the prostate gland, which can lead to serious health issues if not detected and treated early^2^. The early and accurate diagnosis of PCa is crucial for effective treatment and improved patient prognosis. Magnetic Resonance Imaging (MRI) has become a key tool in the PCa segmentation by offering detailed prostate images^3–5^. PCa segmentation that accurately delineates the tumor boundaries within the prostate in MRI images, is vital for targeted biopsies, personalized treatment planning, and radiation therapy, ensuring that treatments are concentrated on the cancerous tissues while sparing healthy tissues^6^. Additionally, accurate PCa segmentation aids in monitoring disease progression and evaluating treatment efficacy, leading to better-informed clinical decisions and improved patient outcomes^7–9^.

**Fig. 1:**
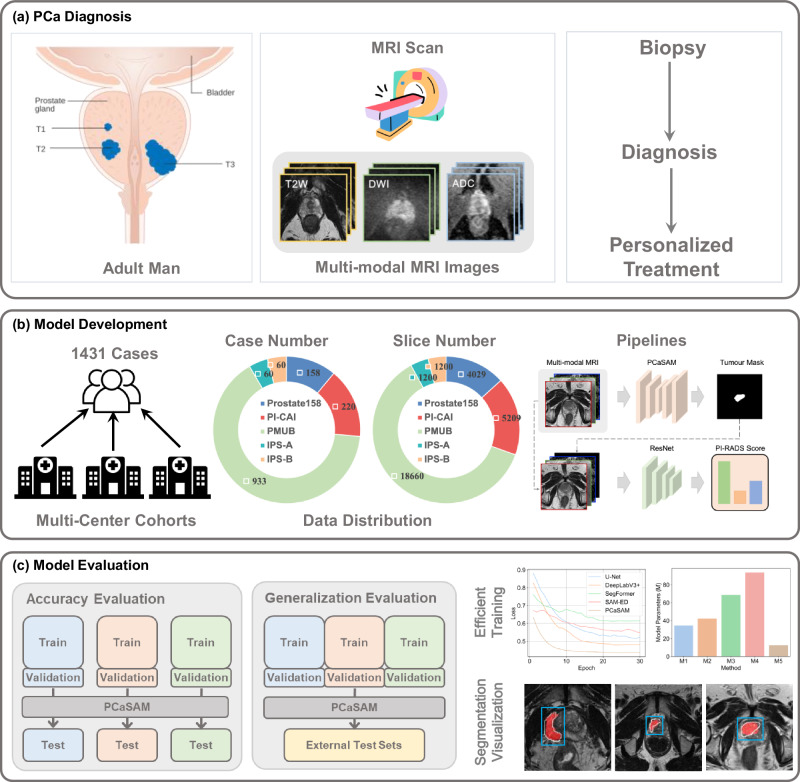
Overview of our study. **a** PCa diagnosis and multi-modal MRI image acquisition, including T2W, DWI and ADC. **b** We constructed multi-center MRI datasets and encompassed 1431 cases in total. We developed a deep-learning model PCaSAM based on the datasets and further utilized the PCa segmentation for PI-RADS scoring. **c** We evaluated the accuracy and generalization of PCaSAM, and presented its efficient training and visualized segmentation results.

Multi-modal MRI imaging is a powerful tool that enhances the diagnosis and assessment of PCa by combining different imaging techniques to provide complementary information^10,11^. T2-weighted (T2W) imaging is a fundamental MRI modality that offers high-resolution images with excellent anatomical detail, helping to identify structural abnormalities in the prostate gland. Diffusion-weighted imaging (DWI) measures the diffusion of water molecules within tissue, providing insights into cellular density. In DWI images, areas with restricted diffusion often indicate tumor presence. Apparent diffusion coefficient (ADC) is derived from DWI and quantifies the degree of water diffusion within tissues. Lower ADC values typically correspond to higher cellular density, which is indicative of malignancy. Together, T2W, DWI and ADC images provide a comprehensive view of the prostate, enhancing the ability to detect, localize and characterize PCa, as shown in Fig. 1a. Although MRI techniques are also able to generate other modalities, including dynamic contrast-enhanced (DCE) images or magnetic resonance spectroscopic imaging (MRSI) images, multi-modal MRI images only refer to T2W, DWI and ADC images in this paper.

Manual PCa segmentation is time-consuming and subject to the experience of radiologists. Advancements in deep learning (DL) techniques have significantly improved PCa segmentation in MRI images^12,13^. U-Net architecture^14^ plays a vital role in most DL-based PCa segmentation models. These U-Net based DL models accurately delineate PCa regions, providing consistent and reproducible results^15–18^. Despite these advancements, PCa segmentation remains challenging. Firstly, tumor appearance heterogeneity, artifacts and noise in MRI images complicate the segmentation task. Secondly, the contrast between tumors and prostate gland in MRI images can be subtle. Thirdly, training deep learning models requires a large volume of high-quality annotated data and the need for robust models that generalize well across different patient populations and MRI protocols adds to the complexity.

Foundation models represent a groundbreaking advancement in medical image analysis, offering transformative potential for various clinical applications^19^, including disease detection^20,21^, organ segmentation^22,23^, reports generation^24,25^, visual question answering^26,27^, etc. These models are pre-trained on vast amounts of diverse data and could be fine-tuned for specific tasks, providing a robust and adaptable solution. Their ability to generalize across different imaging modalities and anatomical structures makes them highly effective for most of medical images. One significant advantage of foundation models is their capacity to leverage transfer learning, allowing them to apply knowledge gained from one domain to another, thus enhancing performance even with limited annotated data^19^. This is particularly beneficial in the medical field, where high-quality labeled datasets are often scarce. The use of foundation models in medical imaging is still in its early stages, but the results so far are promising.

MedSAM^28^ is the medical foundation model supporting universal medical image segmentation, which was developed on a large-scale medical image dataset based on the Transformer architecture^29,30^ of segment anything model (SAM)^31^. MedSAM is a semi-automated medical segmentation model requiring users to draw a bounding box manually as the prompt to guide the mask decoder. Although MedSAM presents remarkable performance for most of medical image segmentation tasks, there are still some limitations. Firstly, MedSAM processes single-modality 3-channel images but cannot fuse multi-modal medical images for improved image segmentation. Secondly, MedSAM is sensitive to the bounding boxes provided by users. Thirdly, MedSAM was mainly trained for organ segmentation in medical images with different modalities and it remains challenging for the difficult PCa segmentation.

In this study, we developed and validated PCaSAM by integrating multi-modal MRI images and MedSAM. We collected multi-center datasets and conducted a comprehensive evaluation. In our experiments, PCaSAM achieved the highest performance in all the internal datasets and presented powerful generalization ability in the external datasets, highlighting the necessity of multi-modal information integration for PCa segmentation. It also indicates medical foundation models can be successfully applied to address the challenging MRI segmentation problems. PCaSAM is efficient enough, which has fewer trainable parameters and faster convergence speed, which is highly suitable for deployment scenarios. The PCa segmentation further improved the Prostate Imaging Reporting and Data System (PI-RADS) scoring of PCa significantly. PCaSAM assists radiologists by providing accurate and automated PCa segmentation results, indicating potential for real-world usage as a promising image-level assistant tool.

## Results

### Study design

The overview of this work is illustrated in Fig. 1. In this study, we collected multi-center multi-modal MRI PCa segmentation datasets from both public and private sources for our model evaluation, including Prostate158^32^, PI-CAI^33^, PMUB^34^, IPS-A and IPS-B. A total of 1431 cases were collected from multiple clinical centers around the world, where Prostate158 was collected from Charité-Universitätsmedizin Berlin, PI-CAI was collected from Radboud University Medical Center, Ziekenhuis Groep Twente, University Medical Center Groningen, PMUB was collected from The UCLA Health Prostate Cancer Program, IPS-A was collected from Beijing Hospital and IPS-B was collected from The Third Medical Center of Chinese PLA General Hospital. In PMUB, lesions were included only if ISUP≥2 was confirmed by histopathology and ISUP 1 lesions were excluded. Radiologists delineated masks on these biopsy-confirmed lesions. In contrast, Prostate158 and PI-CAI data relied on consensus radiology reads, and IPS-A/B on expert radiologist PI-RADS annotations. Segmentation metrics are computed based on their segmentation masks.

Each case contained multi-modal MRI volumes, namely T2W, DWI and ADC volumes. PI-CAI dataset was misaligned due to multiple factors in the scanning process, we applied a two-stage registration on it. Specifically, we first annotated 8 anatomical landmarks across multi-modal MRI images and then used a deformable B-spline method for registration. The resulting deformation fields were applied both to the images and the lesion masks. This ensures that our segmentation models operate on well-aligned multi-modal MRI images, mitigating the need to learn invariance to misregistration. Besides, we visually inspected samples and found sub-voxel alignment for other datasets, including Prostate158, PMUB, IPS-A and IPS-B. Therefore, we did not perform additional registration on Prostate158, PMUB, IPS-A, or IPS-B. Finally, all multi-modal MRI images were aligned in the physical space. The number of cases and MRI slices of datasets are presented in Fig. 1b. To ensure the preciseness of our study, two senior radiologists were invited to review, validate and correct the annotations of datasets to reduce the gap between what constitutes a positive lesion among all datasets.

In our experiments, Prostate158, PI-CAI and PMUB were chosen as the internal datasets, and IPS-A and IPS-B were left as the external datasets. We trained and assessed the segmentation performance of PCaSAM against existing segmentation models using the internal datasets individually. In other words, we split each internal dataset into the training, validation and testing set with a ratio of 7:1:2, and then evaluated the testing set by the model trained on the training set from the corresponding dataset, as shown in Fig. 1c. The validation set was used to select the best model parameters during the training phase. We also conducted other exploratory and interpretive research based on the internal datasets, including the ablation studies and exploring the modality importance. The generalization ability of the model was evaluated on the external datasets by the model trained on the training sets of all the internal datasets, as shown in Fig. 1c. All 3D volumes were split into 2D slices along the axis dimension, leading to 30298 2D multi-modal MRI triples.

### Accurate and generalizable PCa segmentation by PCaSAM

We compared PCaSAM with the medical segmentation foundation model MedSAM and other representative specialist segmentation models, including convolution-based models (U-Net^14^, DeepLabV3+^35^) and Transformer-based models (SegFormer^36^, SAM-ED). SAM-ED is a prompt-free segmentation model that uses the same image encoder and mask decoder as MedSAM but removes the prompt encoder. For all specialist segmentation models, we trained them from scratch without using pre-trained parameters. For MedSAM, we test it directly across all tasks with fixed parameters. Besides, T2W, DWI and ADC images were stacked along the channel dimension to form the three-channel multi-modal inputs for all comparative methods. We selected the dice similarity coefficient (DSC), the most widely used metric for segmentation performance evaluation. DSC was computed globally based on a whole dataset.

**Fig. 2:**
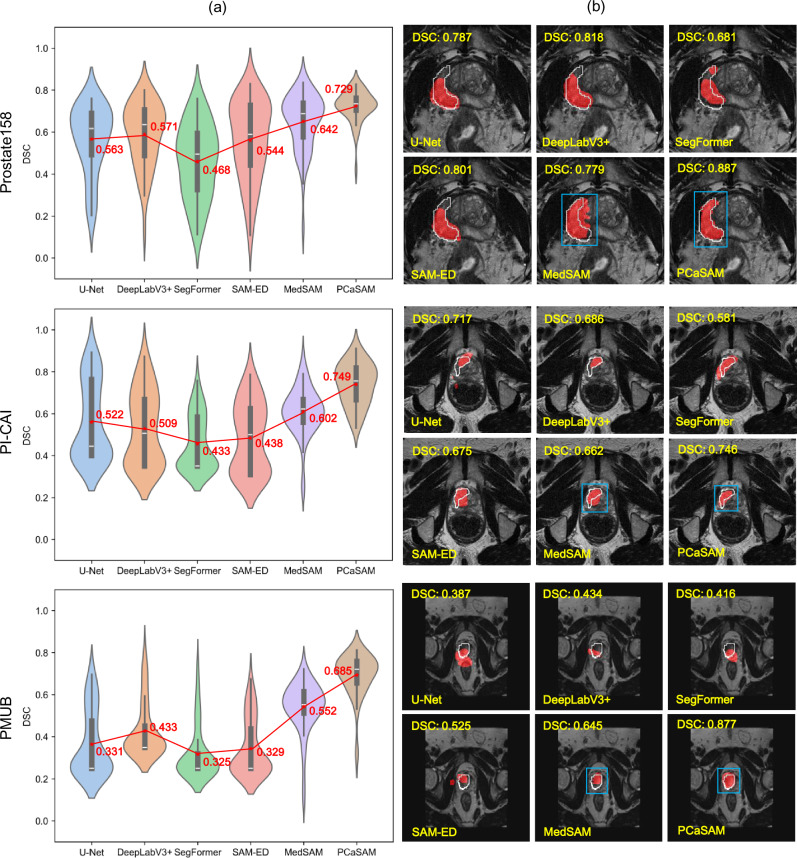
Quantitative and qualitative evaluations on three internal datasets. **a** Violin plot presents the DSC distributions of all methods. Wider sections of the violin represent a higher density of data points, while narrower sections represent a lower density. The white line within the box represents the median value, with the bottom and top bounds of the box delineating the 25th and 75th percentiles, respectively. The red star within the box represents the average value. **b** Visualized segmentation examples. Blue: bounding box prompts; Red: segmentation results; White: expert annotations.

Figure  2a displays the DSC distributions for all methods across three internal datasets, and the source data is provided in Supplementary Table 1. Overall, SegFormer achieved the lowest performance across all internal datasets (Prostate158: 0.468, PI-CAI: 0.433, PMUB: 0.325). Although the other three specialist segmentation models outperformed SegFormer, their improvements were marginal. Two convolution-based methods performed better than the other two Transformer-based methods. MedSAM showed a narrower DSC distribution across all the internal datasets. PCaSAM achieved the best performance (Prostate158: 0.729, PI-CAI: 0.749, PMUB: 0.685), increasing by average 8.7%–14.7% in absolute terms respectively on three datasets compared to MedSAM, improving PCa segmentation performance. Figure 2(b) visualizes randomly selected segmentation examples from the three internal datasets. Here, we stacked the segmented PCa masks onto T2W images for a clearer qualitative visualization. All comparative methods struggled with weak boundaries, often resulting in under- or over-segmentation errors. In contrast, PCaSAM accurately segments a various range of PCa under various imaging conditions, achieving better performance than the other methods.

**Fig. 3:**
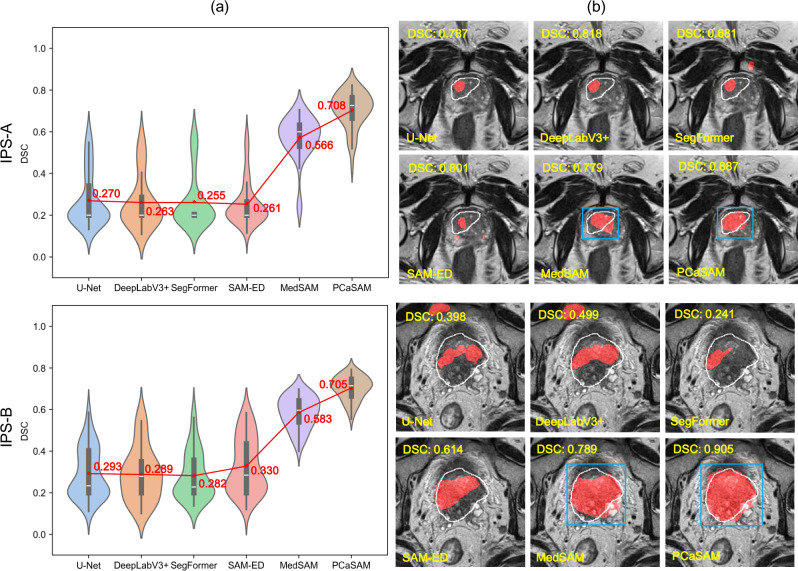
Quantitative and qualitative evaluation results on two external datasets. **a** Violin plot presents the DSC distributions of all methods. **b** Visualized segmentation examples. Blue: bounding box prompts; Red: segmentation results; White: expert annotations.

In addition, we evaluated PCaSAM’s generalizability by conducting cross-dataset validations. Specifically, we trained the segmentation models on the combined training sets of three internal datasets and tested it on two external datasets. The quantitative results are shown in Fig. 3a, an obvious decline of the average DSC is observed among all the specialist segmentation models when transitioning from the internal datasets to two external datasets. Compared to specialist segmentation models such as U-Net (from 0.472 to 0.270), DeepLabV3+ (from 0.504 to 0.278), SegFormer (from 0.409 to 0.263) and SAM-ED (from 0.437 to 0.295), MedSAM (from 0.598 to 0.575) and PCaSAM (from 0.721 to 0.706) have a smaller alteration, with merely 0.023 and 0.015 decreases of DSC respectively, suggesting that foundation model-based approaches have superior generalization ability. Additionally, PCaSAM reduced the standard deviation of performance across datasets, indicating increased stability in diverse imaging scenarios. Figure 3(b) visualizes randomly selected segmentation examples from two external datasets. As observed, for the cross-dataset validations, specialist segmentation models showed more severe under- or over-segmentation errors, while MedSAM and PCaSAM obtained more stable PCa segmentation results and PCaSAM performed better than MedSAM. These results suggested that PCaSAM not only excels in precision but also provides more robust segmentation in the presence of varying imaging conditions, positioning it as a more versatile tool for clinical applications.

### Efficient training for specific tasks

**Fig. 4:**
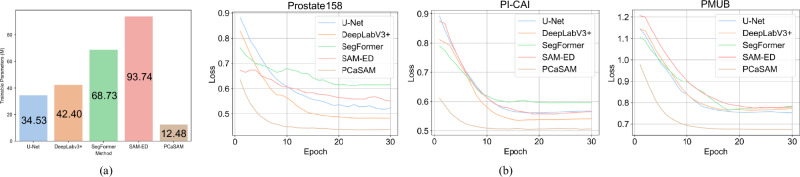
Efficient training for PCaSAM. (**a**) compares the number of trainable parameters between PCaSAM and other specialist segmentation models. PCaSAM has the least trainable parameters (12.48 M), only about 36% of U-Net. (**b**) compares the convergence speed on three internal datasets. PCaSAM converges quickly within 5 epochs, while other specialist models need at least about 15 epochs.

PCaSAM demonstrated its efficiency in reducing time and hardware constraints. It keeps the parameters of the pre-trained image encoder, prompt encoder, and mask decoder fixed, while only the two multi-modal fusion modules (MFMs) require optimization during training. Each MFM has 6.24 M parameters, leading to a total of 12.48 M trainable parameters (MedSAM’s 93.74 M parameters are frozen), just around 36% of those in U-Net (i.e., 34.53 M), while PCaSAM still outperforms U-Net. Figure 4a shows the number of trainable parameters of all specialist models and PCaSAM. We also plotted the convergence curves of all these models across three internal datasets, as shown in Fig. 4b. PCaSAM demonstrated faster convergence speed than other specialist models, specifically reaching global optimization within 5 epochs, whereas other specialist models require at least 15 epochs.

We further compared the required VARM during training. The prompt-free segmentation network had 726.3M VARM and the prompt-guided segmentation network had 747.4M VARM when the batch size was set to 1. Both had fewer VARM than U-Net (770.7M). Besides, the wall time from our proposed two segmentation networks and U-Net are about 6.8h, 7.3h and 8.6h when we trained them on the combination of training sets of three internal datasets. PCaSAM had slightly lower VRAM requirement during training, which is able to be trained and deployed on an 8 GB VRAM GPU, while requiring more during inference, and was slower than U-Net.

### PI-RADS scoring based on PCa segmentation

The Prostate Imaging Reporting and Data System (PI-RADS) scoring is essential for radiologists to evaluate the severity of PCa in clinical scenarios. Based on the PCa segmentation, it is easier to focus on the PCa status and evaluate the PI-RADS score of each PCa lesion. In general, the severity of PCa can be scored into 5 levels, and their brief descriptions are illustrated in Supplementary Table 5. Since the PCa lesions with a PI-RADS score of ≤2 are often not clinically reported due to low suspicion of malignancy, the DL-based PI-RADS scoring task aims to perform a classification for PCa lesions with a PI-RADS score of ≥3. We evaluated the PI-RADS scoring performance on IPS-A and IPS-B datasets, which include the PCa lesions with a PI-RADS score of ≥3. In these two datasets, apart from the delineation of the PCa lesions, the corresponding PI-RADS score was further given by a senior radiologist who has 18 years of MRI experience in PCa diagnosis. Another senior radiologist who has 16 years of MRI experience in PCa diagnosis checked and confirmed the annotation results. We split IPS-A and IPS-B for training (30 cases), validation (10 cases) and testing (20 cases).

Although the existing works were built based on the raw MRI images directly^37,38^, we hypothesize that PCa segmentation is able to effectively improve the PI-RADS scoring performance of DL-based methods. Following the general clinical routine, we selected the MRI slice with the largest PCa lesion from each volume for PI-RADS scoring based on the predicted segmentation results. In our study, we selected ResNet50 as the scoring baseline and provided three different versions of model inputs for comparisons. In version 1, we stacked the multi-modal MRI images without PCa segmentation as a three-channel input of ResNet50, called “ResNet50 w/o Masks”, and then ResNet50 predicted the PI-RADS scores. In version 2, we further stacked the PCa segmentation from PCaSAM to the three-channel input of version 1 to form the four-channel model input, called “ResNet50 w/ OurMasks”. In version 3, we further stacked the PCa segmentation from expert annotations to the three-channel input of version 1 to form the four-channel model input, called “ResNet50 w/ GTMasks”. We reported the average Area Under Curve (AUC) for PI-RADS scores of 3, 4 and 5 respectively in Table 1. When we computed the AUC for a specific positive class, the other classes were combined as negative class. After adding the PCa segmentation to the model input for the region’s attention, the scoring performance of ResNet50 on three PI-RADS scores increases in average AUC by 8.3 -8.9% in absolute terms respectively on two external datasets by comparing v1 and v2 Besides, with the groundtruth annotation as the prior knowledge, v3 achieved the best classification performance, but presents no statistically significance over v2, suggesting that PCaSAM has high consistency with the expert annoations.

**Table 1:** Comparison of PI-RADS scoring among three different input versions by the average Area Under Curve (AUC) metric

Methods	IPS-A			IPS-B		
	3	4	5	3	4	5
v1: ResNet50 w/o Masks	0.613	0.544	0.688	0.638	0.548	0.707
v2: ResNet50 w/ OurMasks^†^	0.682	0.586	0.825	0.722	0.603	0.836
v3: ResNet50 w/ GTMasks^†,*^	0.714	0.613	0.866	0.729	0.607	0.843

The Wilcoxon signed-rank test and Benjamini-Hochberg (BH) correction were performed for the results of each target. ^†^indicates statistically significant superiority over v1 (*p* < 0.01, FDR < 0.01), while * denotes statistically non-significant superiority over v2 (*p* > 0.05, FDR < 0.05).

## Discussion

PCa segmentation is more challenging than prostate segmentation in MRI images. Besides the inherent appearance of PCa in MRI images, additional factors such as domain shift and cancer severity significantly increase the difficulty of PCa segmentation. While some existing models have achieved high performance (DSC > 0.7) for PI-RADS 5 lesions^15,16^, lesions with PI-RADS scores ≤4 are more common in clinical scenarios. End-to-end prompt-free models struggle to achieve a DSC > 0.6 for the lesions with PI-RADS score ≤4. For the medical segmentation foundation model MedSAM, despite its effective generalization in organ segmentation, PCa segmentation remains highly challenging. This paper presents PCaSAM, a model designed for accurate, automated and efficient PCa segmentation from multi-modal MRI scans. By comparing “PCaSAM” in Supplementary 2 and “Adaptor-Tuning” in Supplementary Table 2, prompt-guided PCaSAM outperforms its prompt-free counterparts by an average of 13.7% on internal and 13.3% on external datasets. Introducing the bounding box as a prompt significantly improves segmentation accuracy, highlighting the importance of prompts. However, radiologists are burdened by manually drawing bounding boxes in clinical scenarios, making fully automated models more appealing than semi-automated ones.

We confirm that multi-modal MRI images aid radiologists in diagnosing PCa, but we seek to determine whether they indeed enhance DL-based segmentation models and which modality contributes the most. We use the specialist U-Net model to investigate this issue and combine various MRI modalities as input. The results are presented in Supplementary Table 3. By and large, there is an obvious upward trend in segmentation performance when input modalities are increased, from 0.286–0.318 to 0.397–0.412, and further to 0.472. More specifically, for single MRI modality, ADC achieved the best segmentation results (0.318), which is higher than T2W (0.286) and DWI (0.291). For dual-modal input, “DWI+ADC” achieved the best segmentation results (0.433). Lastly, three-modal input “T2W+DWI+ADC” achieved a DSC of 0.472, higher than the single-modal and dual-modal input. Such observations indicate that a combination of T2W, DWI and ADC images brings the maximum useful information for cancer segmentation, in which ADC plays a primary role, while DWI and T2W provide complementary information.

In terms of clinical impact, PCaSAM’s fully automated characteristic reduces the reliance on manual intervention, easing the burden on radiologists and streamlining the diagnostic workflow. By optimizing only the multi-modal fusion modules, PCaSAM achieves state-of-the-art performance with fewer trainable parameters, making it resource-efficient and accessible for institutions with limited computational resources. The accelerated convergence of the model also reduces training times, further emphasizing its efficient deployment for real-world applications. We also evaluate the effectiveness of each component by ablation study in Supplementary Table 4.

While PCaSAM demonstrates strong performance, we acknowledge certain limitations. Firstly, PCaSAM is limited to three MRI modalities due to MedSAM’s three-channel input restriction. Thus, identifying the most important modality as the *query* in the multi-modal fusion module is essential for expanding the number of modalities. Secondly, a noticeable gap persists between automatically generated bounding boxes and expert-drawn ones. The automated bounding box generation module is based on a pre-trained prompt-free segmentation model, limiting it to the model’s segmentation performance. While we implemented a box iterative refinement strategy to get a higher-quality bounding box, it did not reach the upper limits of segmentation performance. Thirdly, while histology provides a more accurate reference, radiology-only datasets inevitably introduce label noise that may attenuate model performance. Our unified model approaches leverage pathology-confirmed diversity (PMUB) and broader scanner/protocol variation (Prostate158, PI-CAI, IPS-A, IPS-B) but remain limited by mixed reference standards. Lastly, in our segmentation evaluation, DSC was computed globally based on a whole dataset. This may mean that large lesions may influence the DSC and small lesions contribute a little. It is more reasonable to be done at the lesion level. Future work will focus on expanding the model’s capability to incorporate additional MRI modalities and refining the automated bounding box generation process to close the performance gap with expert annotations.

In conclusion, this study investigates combining multi-modal MRI images with a medical segmentation foundation model to significantly enhance PCa segmentation performance. PCaSAM, as an accurate and generalized PCa segmentation model, integrating it into a broader clinical pipeline holds substantial potential to revolutionize PCa diagnosis and treatment by providing more reliable and efficient segmentation solutions. Its ability to integrate multi-modal MRI images and leverage advanced deep learning techniques allows for improved accuracy in detecting and delineating lesions, particularly for those with lower PI-RADS scores, which are more frequent and pose greater diagnostic challenges. Overall, the introduction of PCaSAM sets a new benchmark for PCa segmentation, showcasing the advantages of combining multi-modal MRI data with advanced segmentation models. Its adaptability, efficiency, and potential for further development make it a promising tool for enhancing PCa care and improving patient outcomes through timely and targeted interventions, ultimately contributing to the advancement of personalized medicine in oncology.

## Methods

### Foundation model for image segmentation

A large number of foundation models have been published for a variety of computer vision tasks, but most of them rely on the interaction of images and texts^20,22,24–26^. We selected Segment Anything Model (SAM)^31^ as the basis of our study, which is the most popular foundation model for image segmentation and requires no texts as additional guidance. SAM is built on Transformer architecture^29^, which contains an image encoder for mapping the input images into high-dimensional image embedding, a prompt encoder for transforming the user interactions into feature representation, and a mask decoder for generating segmentation probability map by fusing the image embedding, prompt features and output tokens using cross-attention strategy. SAM provides three prompts for the prompt encoder: points, bounding boxes and masks.

Ma et al. followed the Transformer architecture in SAM and presented MedSAM^28^ for medical image segmentation by fine-tuning SAM on a diverse and large-scale medical image segmentation dataset. This dataset contains 1,570,263 medical image-mask pairs covering a multitude of imaging modalities and imaging protocols. Compared to SAM, MedSAM simplified the architecture to better adapt to medical image tasks. Firstly, MedSAM employed the base ViT architecture^30^ as the image encoder to balance the segmentation performance and computational efficiency. Because larger ViT versions offered only marginal improvements in accuracy while significantly increasing computational demands. Secondly, MedSAM removed points and masks from the prompt encoder and only left the bounding boxes as the prompt. Bounding boxes provide a more unambiguous spatial context for the region of interest, enabling the segmentation model to more precisely recognize the target area. Moreover, drawing a bounding box is efficient, especially in multi-object segmentation scenarios.

The image encoder with the base ViT architecture consists of 12 transformer layers, with each block comprising a multi-head self-attention block^29^ and a multilayer perceptron (MLP) block incorporating layer normalization^39^. The input image ***x*** is converted into the image embeddings ***f*** after passing through the image encoder ${\mathcal{E}}(\cdot )$. The prompt encoder ${\mathcal{P}}(\cdot )$ maps the two corner points (top-left and bottom-right) ***b*** of the bounding boxes to a prompt feature ***f***_*p*_. The mask decoder ${\mathcal{D}}(\cdot )$ consists of two Transformer layers for fusing the image embedding and prompt feature, and two transposed convolutional layers to enhance the embedding resolution to 256 × 256. Subsequently, the embedding undergoes Sigmoid activation to obtain the PCa segmentation probability map $\hat{{\boldsymbol{y}}}$, followed by an Otsu method^40^ with a threshold of 0.5 to predict the lesion candidates. The whole segmentation process in MedSAM can be represented as:

1 $$\begin{array}{lll}{\boldsymbol{f}}&=&{\mathcal{E}}({\boldsymbol{x}}),\,\,{\rm{where}}\,{\boldsymbol{x}}\in {{\mathbb{R}}}^{1024\times 1024\times 3},\,{\boldsymbol{f}}\in {{\mathbb{R}}}^{64\times 64\times 256}\\ {{\boldsymbol{f}}}_{p}&=&{\mathcal{P}}({\boldsymbol{b}}),\,\,{\rm{where}}\,{\boldsymbol{b}}\in {{\mathbb{R}}}^{4},\,{{\boldsymbol{f}}}_{p}\in {{\mathbb{R}}}^{256}\\ \hat{{\boldsymbol{y}}}&=&{\mathcal{D}}({\boldsymbol{f}},{{\boldsymbol{f}}}_{p}),\,\,{\rm{where}}\,\hat{{\boldsymbol{y}}}\in {{\mathbb{R}}}^{256\times 256}\end{array}$$

In general, users must draw the bounding box ***b*** manually, leading to the semi-automated segmentation model.

### Overview of PCaSAM

**Fig. 5:**
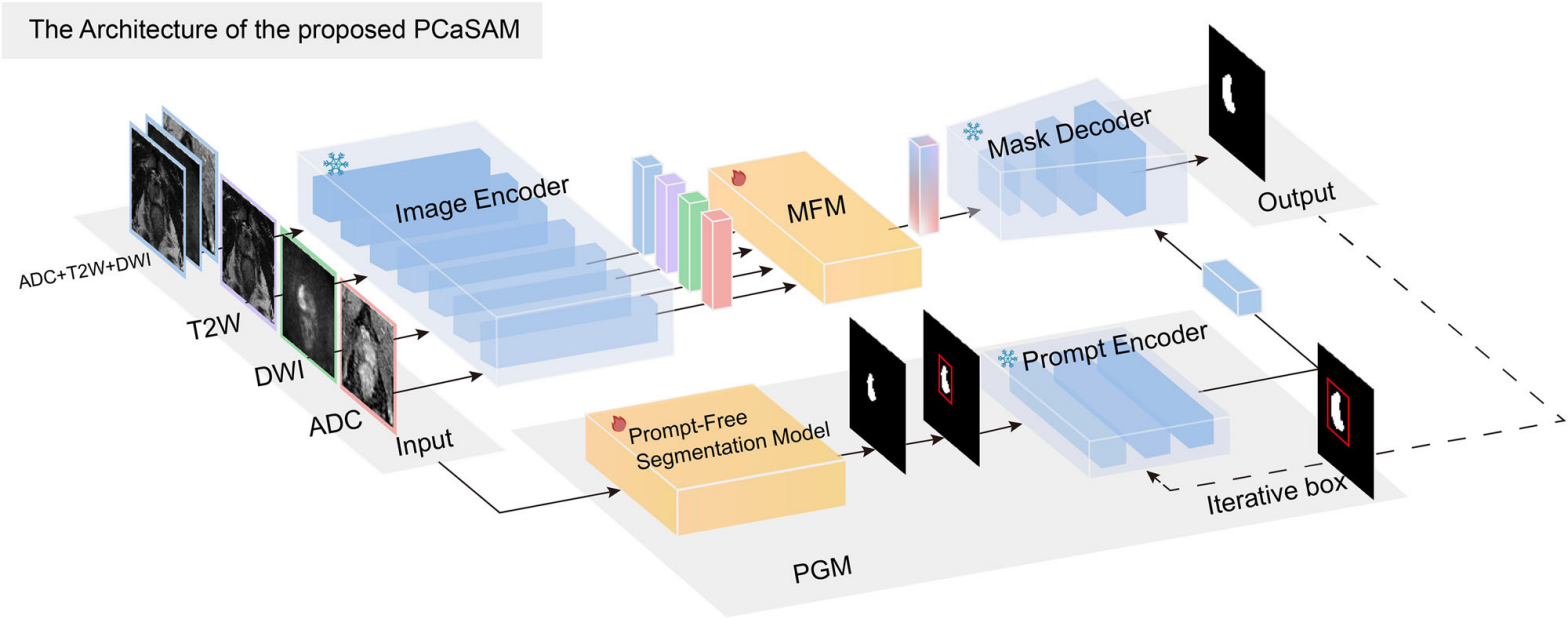
Overview of the proposed PCaSAM. Multi-modal MRI images are sent into image encoder to obtain the corresponding multi-modal features, and then the multi-modal fusion module (MFM) combines the multi-modal features for the mask decoder. Besides, the prompt generation module (PGM) generates the bounding boxes automatically for the guidance. The snowflake symbol denotes the parameters of modules are frozen, and the flame symbol denotes the parameters of modules are optimized.

Figure 5 shows the overview of PCaSAM architecture. PCaSAM first transfers MedSAM’s pre-trained image encoder, prompt encoder and mask decoder. Then PCaSAM adds a Multi-modal Fusion Module (MFM) between the image encoder and mask decoder, and a Prompt Generation Module (PGM) to replace the manual interaction for automated bounding box generation.

Given a group of single-channel multi-modal MRI images {***x***_*t*_, ***x***_*d*_, ***x***_*a*_}, where ${{\boldsymbol{x}}}_{t}\in {{\mathbb{R}}}^{1024\times 1024}$ is the T2W image, ${{\boldsymbol{x}}}_{d}\in {{\mathbb{R}}}^{1024\times 1024}$ is the DWI image and ${{\boldsymbol{x}}}_{a}\in {{\mathbb{R}}}^{1024\times 1024}$ is the ADC image, all of whom are aligned in the physical space. We pass them through the image encoder to obtain the corresponding image embeddings:

2 $${{\boldsymbol{f}}}_{all}={\mathcal{E}}([{{\boldsymbol{x}}}_{a},{{\boldsymbol{x}}}_{d},{{\boldsymbol{x}}}_{t}]),\,{{\boldsymbol{f}}}_{t}={\mathcal{E}}({{\boldsymbol{x}}}_{t}),\,{{\boldsymbol{f}}}_{d}={\mathcal{E}}({{\boldsymbol{x}}}_{d}),\,{{\boldsymbol{f}}}_{a}={\mathcal{E}}({{\boldsymbol{x}}}_{a})$$

where [***x***_*a*_, ***x***_*d*_, ***x***_*t*_] denotes stacking ***x***_*a*_, ***x***_*d*_ and ***x***_*t*_ in the channel dimension. Because the image encoder in MedSAM is fixed to receive the 3-channel inputs, we stack the same image three times in the channel dimension to obtain the 3-channel inputs for single-modal MRI images. Then, MFM combines them to generate the fused embedding ${{\boldsymbol{f}}}^{{\prime} }$. Finally, the fused embedding and the prompt feature from PGM are sent into the mask decoder to obtain improved PCa segmentation results. Compared to Eq. (1), PCaSAM is extended as follows by adding MFM and PGM:

3 $$\begin{array}{l}{{\boldsymbol{f}}}_{all},{{\boldsymbol{f}}}_{t},{{\boldsymbol{f}}}_{d},{{\boldsymbol{f}}}_{a}={\rm{Eq}}.(2)\\ {{\boldsymbol{f}}}_{p}={\mathcal{P}}({\boldsymbol{b}}),\,\,{\boldsymbol{b}}={\rm{PGM}}({{\boldsymbol{x}}}_{t},{{\boldsymbol{x}}}_{d},{{\boldsymbol{x}}}_{a})\\ {{\boldsymbol{f}}}^{{\prime} }={\rm{MFM}}({{\boldsymbol{f}}}_{all},{{\boldsymbol{f}}}_{t},{{\boldsymbol{f}}}_{d},{{\boldsymbol{f}}}_{a})\\ \hat{{\boldsymbol{y}}}={\mathcal{D}}({{\boldsymbol{f}}}^{{\prime} },{{\boldsymbol{f}}}_{p})\end{array}$$

During training, only MFM( ⋅ ) and PGM( ⋅ ) are optimized and all parameters (${\mathcal{E}}(\cdot )$, ${\mathcal{P}}(\cdot )$, ${\mathcal{D}}(\cdot )$) from MedSAM are frozen for preserve the generalization ability of the foundation model.

### Attention for Multi-modal Fusion

**Fig. 6:**
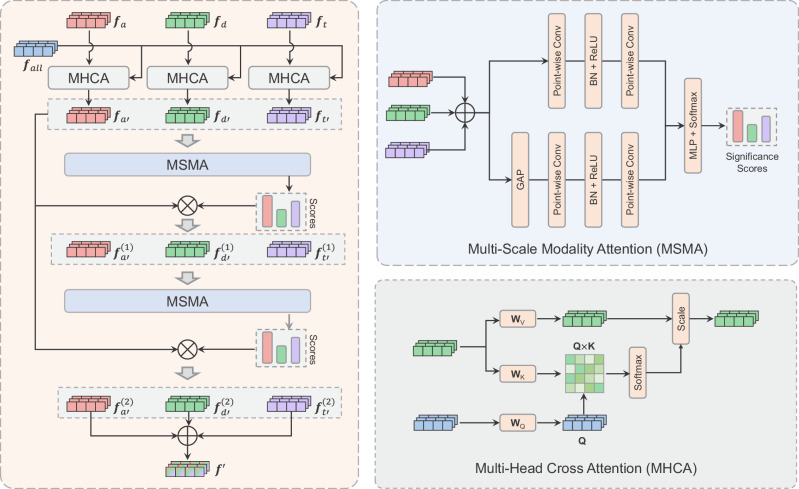
Details of multi-modal fusion module (MFM). Multi-modal features from the image encoder are combined together in MFM by two main components MHCA and MSMA.

Multi-modal fusion module (MFM) contains two key attention methods, multi-head cross-attention (MHCA) for modal interaction and multi-scale modality attention (MSMA) for modal fusion, as shown in Fig. 6.

In MFM, given ***f***_*all*_, ***f***_*t*_, ***f***_*d*_ and ***f***_*a*_ from the image encoder, we provide three MHCA blocks for ***f***_*t*_, ***f***_*d*_, ***f***_*a*_ respectively. In each MHCA block, we regard ***f***_*all*_ as the query feature, and {***f***_*a*_, ***f***_*d*_, ***f***_*t*_} as the key feature and the value feature, to learn PCa-related context from different modalities and then generate three new features:

4 $$\begin{array}{rcl}{{\boldsymbol{f}}}_{{t}^{{\prime} }}&=&{{\rm{MHCA}}}^{(1)}(query={{\boldsymbol{f}}}_{all},\,key={{\boldsymbol{f}}}_{t},\,value={{\boldsymbol{f}}}_{t})\\ {{\boldsymbol{f}}}_{{d}^{{\prime} }}&=&{{\rm{MHCA}}}^{(2)}(query={{\boldsymbol{f}}}_{all},\,key={{\boldsymbol{f}}}_{d},\,value={{\boldsymbol{f}}}_{d})\\ {{\boldsymbol{f}}}_{{a}^{{\prime} }}&=&{{\rm{MHCA}}}^{(3)}(query={{\boldsymbol{f}}}_{all},\,key={{\boldsymbol{f}}}_{a},\,value={{\boldsymbol{f}}}_{a})\end{array}$$

The specific theory about MHCA please refers to the literature^29^.

MSMA implements channel attention on global and local scales by varying the spatial pooling size. Followed by MHCA, a two-stage MSMA with residual connection is used to fuse ${{\boldsymbol{f}}}_{{t}^{{\prime} }}$, ${{\boldsymbol{f}}}_{{d}^{{\prime} }}$ and ${{\boldsymbol{f}}}_{{a}^{{\prime} }}$ by computing their significance score. The specific process is represented as follows:

5 $$\begin{array}{ll}{\rm{Stage(1)}}:\,&{{\boldsymbol{f}}}_{{t}^{{\prime} }}^{(1)}={\alpha }^{(1)}{{\boldsymbol{f}}}_{{t}^{{\prime} }},\,{{\boldsymbol{f}}}_{{d}^{{\prime} }}^{(1)}={\beta }^{(1)}{{\boldsymbol{f}}}_{{d}^{{\prime} }},\,{{\boldsymbol{f}}}_{{a}^{{\prime} }}^{(1)}={\gamma }^{(1)}{{\boldsymbol{f}}}_{{a}^{{\prime} }}\\ &{\alpha }^{(1)},\,{\beta }^{(1)},\,{\gamma }^{(1)}={{\rm{MSMA}}}^{(1)}({{\boldsymbol{f}}}_{{t}^{{\prime} }}+{{\boldsymbol{f}}}_{{d}^{{\prime} }}+{{\boldsymbol{f}}}_{{a}^{{\prime} }})\\ {\rm{Stage(2)}}:\,&{{\boldsymbol{f}}}_{{t}^{{\prime} }}^{(2)}={\alpha }^{(2)}{{\boldsymbol{f}}}_{{t}^{{\prime} }},\,{{\boldsymbol{f}}}_{{d}^{{\prime} }}^{(2)}={\beta }^{(2)}{{\boldsymbol{f}}}_{{d}^{{\prime} }},\,{{\boldsymbol{f}}}_{{a}^{{\prime} }}^{(2)}={\gamma }^{(2)}{{\boldsymbol{f}}}_{{a}^{{\prime} }}\\ &{\alpha }^{(2)},\,{\beta }^{(2)},\,{\gamma }^{(2)}={{\rm{MSMA}}}^{(2)}({{\boldsymbol{f}}}_{{t}^{{\prime} }}^{(1)}+{{\boldsymbol{f}}}_{{d}^{{\prime} }}^{(1)}+{{\boldsymbol{f}}}_{{a}^{{\prime} }}^{(1)})\\ {\rm{Fusion}}:\,&{{\boldsymbol{f}}}^{{\prime} }={{\boldsymbol{f}}}_{{t}^{{\prime} }}^{(2)}+{{\boldsymbol{f}}}_{{d}^{{\prime} }}^{(2)}+{{\boldsymbol{f}}}_{{a}^{{\prime} }}^{(2)}\end{array}$$

where *α*^(1)^, *β*^(1)^, *γ*^(1)^, *α*^(2)^, *β*^(2)^, *γ*^(2)^ are the significance scores for different modality, ${{\boldsymbol{f}}}^{{\prime} }$ is the final fused embedding.

### Prompt-free segmentation for prompt generation

A fully automated PCa segmentation model is preferred by clinicians, so we proposed a prompt generation module (PGM) to replace the manual bounding box. PGM contains three steps, (1) obtaining the coarse PCa segmentation, (2) morphological post-processing, and (3) box iterative refinement. PGM is a segmentation-only module, which takes multi-modal MRI as input and outputs a coarse segmentation mask. There is no intermediate object-detection or bounding-box proposal network. The bounding box is obtained by a morphological post-processing operation on the coarse segmentation mask. PGM is evaluated using segmentation metrics only.

A prompt-free segmentation model is used to obtain a coarse PCa segmentation. The prompt-free segmentation model could be any pre-trained end-to-end segmentation model, including FCN^41^, U-Net^14^ or DeepLabV3+^35^. However, training a prompt-free PCa segmentation model from scratch is sub-optimal^42^. To maintain the efficiency, we convert Eq. (3) into the prompt-free segmentation model by removing the prompt encoder:

6 $$\begin{array}{ll}&{{\boldsymbol{f}}}_{all},{{\boldsymbol{f}}}_{t},{{\boldsymbol{f}}}_{d},{{\boldsymbol{f}}}_{a}={\rm{Eq}}.(2)\\ &{{\boldsymbol{f}}}^{{\prime} }={\rm{MFM}}({{\boldsymbol{f}}}_{all},{{\boldsymbol{f}}}_{t},{{\boldsymbol{f}}}_{d},{{\boldsymbol{f}}}_{a})\\ &\hat{{\boldsymbol{y}}}={\mathcal{D}}({{\boldsymbol{f}}}^{{\prime} })\end{array}$$

It is noted that MFM in Eq. (3) and Eq. (6) are not shared. The image encoder and mask decoder in Eq. (6) are fixed, and only one MFM needs to be trained, ensuring low computation costs and fewer training samples, meanwhile protecting the generalization ability of MedSAM.

After obtaining the coarse PCa mask, morphological post-processing uses a morphological open-close operation to remove the discrete outliers from coarse masks and merge the adjacent connected areas. Then we divide the PCa mask with multiple PCa areas into multiple masks with a single connected PCa area.

We compute the minimum enclosing rectangle for each connected area and then expand it outward by 15% as the initial bounding box. Lastly, we refine each bounding box by a box iterative refinement. Specifically, the bounding box and the fused image embedding are used to obtain the refined PCa mask. The refined PCa mask is used to compute the bounding box and continue to obtain the next PCa mask until the DSC metric between the current PCa mask and the previous PCa mask converges. The box iterative refinement can be seen in Algorithm 1.

#### Algorithm 1

Box Iterative Refinement

**Require**:

 Fused Image Embedding ${{\boldsymbol{f}}}^{{\prime} }\in {{\mathbb{R}}}^{64\times 64\times 256}$,

 Initial PCa Mask ${{\boldsymbol{m}}}^{(0)}\in {{\mathbb{R}}}^{256\times 256}$,

 Prompt Encoder $${\mathcal{P}}(\cdot )$$,

 Mask Decoder $${\mathcal{D}}(\cdot )$$.

**Ensure**: Final PCa Mask ${{\boldsymbol{m}}}^{final}\in {{\mathbb{R}}}^{256\times 256}$

 1: obtain the minimum enclosing rectangle from ***m***^(0)^ and expands outward by 15% as the initial bounding box ***b***^(0)^;

 2: Repeat *I* (*I*≥1) times until DSC(***m***^(*I*−1)^, ***m***^(*I*)^)≥0.95:

 (1) $${{\boldsymbol{f}}}_{p}^{(i)}$$ = $${\mathcal{P}}({{\boldsymbol{b}}}^{(i-1)})$$, $${\hat{{\boldsymbol{y}}}}^{(i)}$$ = $${\mathcal{D}}({{\boldsymbol{f}}}^{{\prime} },{{\boldsymbol{f}}}_{p}^{(i)})$$, *i* ∈ [1, *I*];

 (2) obtain the PCa mask ***m***^(*i*)^ based on ${\hat{{\boldsymbol{y}}}}^{(i)}$ by a threshold of 0.5;

 (3) obtain the minimum enclosing rectangle from ***m***^(*i*)^ and expands outward by 10% as the bounding box ***b***^(*i*)^;

 3: ***m***^*final*^ = ***m***^(*I*)^

### Training and implementation details

In our study, the image encoder, prompt encoder and mask decoder were initialized with the pre-trained parameters of MedSAM with the base ViT model, and their parameters were fixed to maintain the powerful generalization of MedSAM. During training, we only needed to optimize two MFMs in the prompt-guided and prompt-free segmentation networks. To get the whole PCaSAM, we first trained the prompt-free segmentation model and then frozen it to train the prompt-guided segmentation model. Both of them were optimized by Adam optimizer (*β*1=0.9, *β*2=0.999) with an initial learning rate of 1e-5 and a weight decay of 0.01. For the prompt-guided segmentation model, the bounding box was simulated from the expert annotations with a random perturbation of 0-20 pixels along the width and height. The global batch size was 8 and the random rotation was used for data augmentation. We resized the T2W, DWI and ADC images to 1024 × 1024 to fit the input size of the image encoder in MedSAM. The model was trained on two NVIDIA 3090 GPUs with 30 epochs and the checkpoints that performed best on the validation set were saved for testing. For comparative specialist segmentation models, we trained them on one NVIDIA 3090 GPU with 30 epochs and the checkpoints that performed best on the validation set were saved for testing. We resized the T2W, DWI and ADC images to 1024 × 1024, and stacked them along the channel to form the three-channel input. Python 3.9 and Pytorch 2.4.0 were used to perform training and inference for all segmentation models. All models that need to be trained from scratch were initialized using Kaiming initialization.

At the end of the mask decoder, we used the unweighted sum between binary cross-entropy loss and dice loss as the final loss function since it has been proven to be robust across different medical image segmentation tasks:

7 $${\mathcal{L}}=\frac{1}{N}\mathop{\sum }\limits_{n=1}^{N}{{\mathcal{L}}}_{BCE}(\hat{{\boldsymbol{y}}},{\boldsymbol{y}})+{{\mathcal{L}}}_{Dice}(\hat{{\boldsymbol{y}}},{\boldsymbol{y}})$$

where $\hat{{\boldsymbol{y}}}$ is the predicted segmentation probability map from the mask decoder, ***y*** is the expert annotations, *N* is the number of samples in a mini-batch.

### Statistical analysis

In our study, we applied the Wilcoxon signed-rank test to evaluate the performance of all PCa segmentation models. We measured the performance using the DSC for each segmented image, resulting in paired samples for both methods. The Wilcoxon signed-rank test, a non-parametric test, was used to compare these paired differences. This test allowed us to determine whether there is a statistically significant difference in these segmentation models, providing a robust evaluation even when the data does not follow a normal distribution. Besides, we further applied the Benjamini-Hochberg (BH) correction for multiple comparisons to control the false discovery rate. BH balances sensitivity and specificity, enhancing statistical power while tolerating limited false discoveries.

## Supplementary information

Supplementary information

## Data Availability

Refs. ^32–34^ provide access to the Prostate158 (https://github.com/kbressem/prostate158), PI-CAI (https://pi-cai.grand-challenge.org/), and PMUB (https://www.cancerimagingarchive.net/collection/prostate-mri-us-biopsy/) datasets supporting this study's findings. IPS-A and IPS-B used in this study are not publicly available due to hospital regulations and patient privacy restrictions. Readers can request IPS-A and IPS-B from the author, Mengling Wu (wumenglin@njtech.edu.cn), for non-commercial purposes. Figure 2a displays the DSC distributions for all methods across three internal datasets, and the source data are provided in Supplementary Table 1.

## Code availability

The codes of the proposed model in this study have been deposited at github (https://github.com/ZhangYH0502/PCaSAM), which can only be used for non-commercial research purposes.
