# Supplementary material for: Generalist medical foundation model improves prostate cancer segmentation from multimodal MRI images
Source: NPJ Digit Med. 2025 Jun 18;8:372. doi: 10.1038/s41746-025-01756-2 (PMC12177055; doi:10.1038/s41746-025-01756-2)
Supplement: Supplementary file 1 — Supplementary information [file 41746_2025_1756_MOESM1_ESM.pdf]

Supplementary Materials

Supplementary Table 1: Quantitative evaluations on internal datasets and external datasets for validating the accuracy and generalization of all methods by mean value±std of DSC scores. The Wilcoxon signed-rank test and Benjamini-Hochberg (BH) correction were performed for the results of each target. † indicates statistically significant superiority over other specialist segmentation models (p <0.01, FDR<0.01), while # denote statistically significant superiority over MedSAM (p >0.05, FDR<0.05).

Methods	Internal Datasets			External Datasets	
	Prostate158	PI-CAI	PMUB	IPS-A	IPS-B
U-Net	0.563±0.174	0.522±0.165	0.331±0.129	0.270±0.117	0.293±0.105
DeepLabV3+	0.571±0.160	0.509±0.170	0.433±0.126	0.263±0.129	0.289±0.112
SegFormer	0.468±0.180	0.433±0.119	0.325±0.122	0.255±0.121	0.282±0.113
SAM-ED	0.544±0.197	0.438±0.147	0.329±0.140	0.261±0.112	0.330±0.137
MedSAM†	0.642±0.149	0.602±0.126	0.552±0.121	0.566±0.115	0.583±0.082
PCaSAM$^{\dagger,\#}$	0.729±0.066	0.749±0.108	0.685±0.094	0.708±0.077	0.705±0.060

1 Fine-tuning the foundation model effectively

Although the medical foundation models have achieved remarkable results for most common medical image tasks, many specific clinical tasks are still not involved in the foundation models. For example, MedSAM cannot achieve the upper bound of PCa segmentation, even if it performs well in prostate organ segmentation. To explore the suitable strategies of fine-tuning a pre-trained medical foundation model, we design additional experiments based on the PCa segmentation task. The medical foundation model MedSAM contains three main components, the image encoder, the mask decoder and the prompt encoder. We remove the prompt encoder and convert the MedSAM into the end-to-end prompt-free segmentation model. Specifically, we evaluate multiple different tuning strategies to excavate the potential of the medical foundation model:

- Encoder-Tuning: fix the mask decoder and fine-tune the image encoder.
- Decoder-Tuning: fix the image encoder and fine-tune the mask decoder.
- Full-Tuning: fine-tune both the image encoder and the mask decoder.
- Adaptor-Tuning: We fix both the image encoder and the mask decoder, and insert trainable adaptors between the image encoder and the mask decoder. In this paper, the inserted adaptor is the MFM.

Supplementary Table 2: Quantitative comparisons on all the datasets.

Methods	# Para. (M)	Internal Datasets			External Datasets	
		Prostate158	PI-CAI	PMUB	IPS-A	IPS-B
Encoder-Tuning	89.67	0.605±0.185	0.543±0.196	0.441±0.217	0.313±0.177	0.307±0.169
Decoder-Tuning	4.07	0.576±0.201	0.526±0.224	0.413±0.233	0.324±0.189	0.318±0.154
Full-Tuning	93.74	0.598±0.176	0.549±0.215	0.425±0.206	0.318±0.182	0.310±0.158
Adaptor-Tuning	6.24	0.653±0.143	0.597±0.151	0.503±0.120	0.559±0.135	0.588±0.126

From the results in Supplementary Table 2, all tuning methods show superior results than the specialist segmentation models in Supplementary Table 1 that were trained from scratch. It indicates that it is necessary to fine-tune the foundation model on your specific task to achieve better

results. Encoder-Tuning, Decoder-Tuning and Full-Tuning present obvious improvements in individual and total evaluation, while performing poorly for cross-dataset evaluations. The reason is that fine-tuning the parameters of MedSAM directly may disturb the foundation model's generalization. Our Adaptor-Tuning not only improve the accuracy of PCa segmentation significantly and protects the original generalization of MedSAM. Besides, our Adaptor-Tuning has far fewer trainable parameters than Encoder-Tuning and Full-Tuning, while presenting better performance.

Supplementary Table 3: Effect validation of multi-modal MRI images to U-Net model. The table is reported by mean value±std of DSC scores.

Multi-Modal MRI	Prostate158	PI-CAI	PMUB	Average
T2W	0.338±0.153	0.320±0.177	0.201±0.148	0.286±0.159
DWI	0.340±0.147	0.325±0.163	0.207±0.151	0.291±0.154
ADC	0.379±0.143	0.351±0.152	0.224±0.137	0.318±0.144
T2W+DWI	0.480±0.138	0.464±0.182	0.293±0.144	0.412±0.155
T2W+ADC	0.453±0.133	0.452±0.166	0.287±0.135	0.397±0.145
DWI+ADC	0.505±0.127	0.487±0.176	0.306±0.131	0.433±0.145
T2W+DWI+ADC	0.563±0.164	0.522±0.165	0.331±0.129	0.472±0.152
Teică's Method [1]	0.582±0.161	0.518±0.171	0.325±0.127	0.475±0.153

2 Evaluating the importance of multi-modal MRI

We confirm that multi-modal MRI images are beneficial for radiologists to diagnose PCa in the clinical scenario, but we want to know whether multi-modal MRI images indeed improve the performance of DL-based PCa segmentation models and which MRI modality contributes the most to the PCa segmentation task. To explore this, we borrow the specialist U-Net model as the basic segmentation model to verify the thought and combine different MRI modalities as the model input. Specifically, we arrange and combine the three MRI modalities, leading to 7 different inputs for U-Net. Supplementary Table 3 shows the comparative results on three internal datasets. When we only use single-modal MRI input for PCa segmentation, ADC obtains the best results with an average DSC of 0.318. For dual-modal MRI

input, "DWI+ADC" achieved the best segmentation results with an average DSC of 0.433. Lastly, three-modal input "T2W+DWI+ADC" achieved a DSC of 0.472, which is higher than the single-modal input and dual-modal input. Therefore, combining T2W, DWI and ADC images significantly improves segmentation, with ADC playing a primary role, while DWI and T2W provide complementary information.

We introduced a non-deep learning method [1] to highlight and visualize the prostate lesions with a high and very high risk of being malignant by overlaying T2W, ADC and DWI images with a simple yet effective image-processing strategy. Teică's method starts from the premise that the lower the signal intensity is on the T2W and ADC images, and the higher the signal intensity is on the DWI sequence, the more suspicious that lesion is. Supplementary Figure 1 shows the multi-modal fusion results from Teică's method on three internal datasets. We observed that Teică's method performs well on Prostate158, but has poor results on the other two datasets. This non-deep learning method heavily relies on the MRI imaging quality and the severity of PCa lesions, leading to unstable results. We also input the fused images from Teică's method into the U-Net model for further segmentation validation. The qualitative results are shown in Supplementary Figure 1 and quantitative results are presented in Supplementary Table 3. Teică's method performed slightly better than the inputs which concatenate multi-modal images directly, but there was no significant difference between them. Teică's method is a kind of input-level fusion method, and our proposed PCaSAM belongs to feature-level fusion methods. Input-level fusion methods usually fuse multi-modal information before the model input with non-deep learning strategies, it does not fully explore the potential correlation between different modal features, but feature-level methods do.

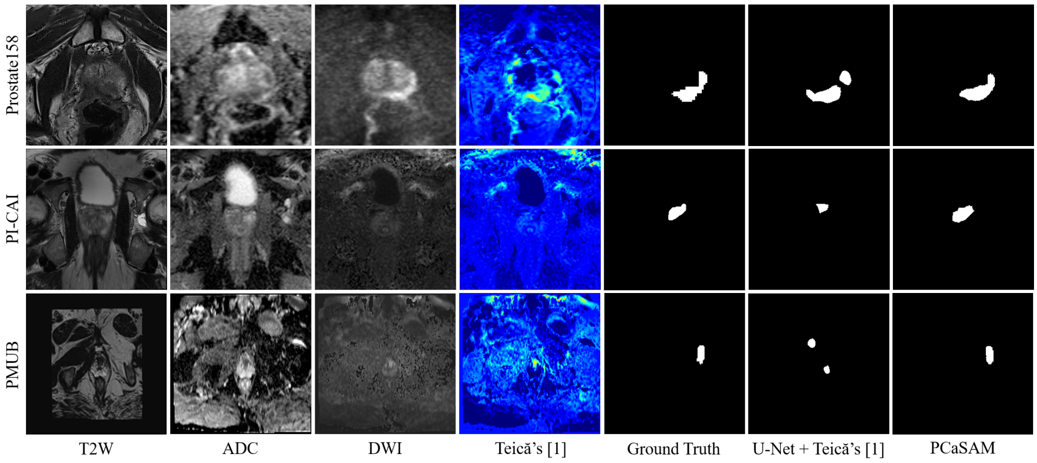

Supplementary Figure 1: **Visual comparison between a non-deep learning method and our PCaSAM.**
Columns 1-3 are the multi-modal MRI images, column 4 shows the fusion results by Teică's method, column 6 shows the segmentation results by inputting the fusion images of column 4, and column 7 shows the results of our proposed PCaSAM.

3 Ablation Study

The effectiveness of the proposed modules is verified by ablation studies on three internal datasets. The performance gain is mainly attributed to two novel modules, the multi-modal fusion module (MFM) and prompt generation module (PGM).

For MFM, we replace it with different feature fusion strategies. The first feature fusion strategy is a three-layer convolutional layers. Multi-modal features from the image encoder are directly concatenated along the channel dimension, and then a three-layer convolutional layers perform the feature fusion and dimension reduction. The second feature fusion strategy is Attentional Feature Fusion (AFF) [2], which aggregates contextual information from different receptive fields for objects of different scales. By comparing (1), (2) and (4) in Supplementary Table 4, our proposed feature fusion strategy presents better performance, which indicates the effectiveness of cross-attention to feature fusion tasks.

PGM consists of three components, namely prompt-free segmentation model, morphological post-processing and box iterative refinement (BIR). Prompt-free segmentation model and morphological post-processing are essential for generating the bounding box automatically. Therefore, we removed BIR to validate its effectiveness. By comparing (3) and (4) in Sup-

plementary Table 4, we can observe that BIR improved the average DSC score of about 1.8-3.4% on three datasets, showing its effectiveness for the refinement of bounding boxes.

Supplementary Table 4: Ablation studies from different module on three internal datasets.

Fusion Method	Prostate158	PI-CAI	PMUB
(1) PCaSAM w/ ConvLayers	0.671±0.144	0.695±0.148	0.625±0.159
(2) PCaSAM w/ AFF	0.688±0.137	0.715±0.141	0.647±0.163
(3) PCaSAM w/o BIR	0.704±0.111	0.731±0.137	0.651±0.123
(4) Our Full PCaSAM	0.729±0.066	0.749±0.108	0.685±0.094

Supplementary Table 5: PI-RADS scoring and their brief descriptions.

Scores	Brief descriptions
1: Very low (highly unlikely cancer)	Clinically significant PCa is highly unlikely. The prostate appears normal, with no suspicious areas on MRI.
2: Low (unlikely cancer)	Findings suggest that clinically significant PCa is unlikely. There may be mild changes in the prostate, but they are not typically associated with malignancy.
3: Intermediate (indeterminate)	Findings are equivocal. Clinically significant PCa is possible but not definite. This score often leads to further clinical evaluation, such as biopsy, for clarification.
4: High (likely cancer)	There is a high likelihood of clinically significant PCa. Abnormal tissue with characteristics of malignancy is present, suggesting that a biopsy or further evaluation is warranted.
5: Very high (highly likely cancer)	Clinically significant PCa is highly likely. MRI shows large, very suspicious areas that are strongly indicative of malignancy, often leading to immediate clinical action like a targeted biopsy.

References

[1] Teică, R. V., Șerbănescu, M.-S., Florescu, L. M. & Gheonea, I. A. Tumor area highlighting using t2wi, adc map, and dwi sequence fusion on bpmri images for better prostate cancer diagnosis. *Life* **13**, 910 (2023).

[2] Dai, Y., Gieseke, F., Oehmcke, S., Wu, Y. & Barnard, K. Attentional feature fusion. In *Proceedings of the IEEE/CVF winter conference on applications of computer vision*, 3560–3569 (2021).
